# Alcohol-attributable mortality and alcohol control policy in the Baltic Countries and Poland in 2001–2020: an interrupted time-series analysis

**DOI:** 10.1186/s13011-023-00574-7

**Published:** 2023-11-09

**Authors:** Ričardas Radišauskas, Mindaugas Štelemėkas, Janina Petkevičienė, Justina Trišauskė, Tadas Telksnys, Laura Miščikienė, Inese Gobina, Relika Stoppel, Rainer Reile, Kinga Janik-Koncewicz, Witold Zatonski, Shannon Lange, Alexander Tran, Jürgen Rehm, Huan Jiang

**Affiliations:** 1 Department of Environmental and Occupational Medicine, Faculty of Public Health, Lithuanian University of Health Sciences, Tilžės Str. 18, 47181 Kaunas, Lithuania; 2 Institute of Cardiology, Lithuanian University of Health Sciences, Sukilėlių Av. 15, 50162 Kaunas, Lithuania; 3 Health Research Institute, Faculty of Public Health, Lithuanian University of Health Sciences, Tilžės Str. 18, 47181 Kaunas, Lithuania; 4 Department of Preventive Medicine, Faculty of Public Health, Lithuanian University of Health Sciences, Tilžės Str. 18, 47181 Kaunas, Lithuania; 5 Department of Public Health and Epidemiology, Riga Stradiņš University, Kronvalda Boulevard 9, Riga, 1010 Latvia; 6 Institute of Public Health, Riga Stradiņš University, Kronvalda Boulevard 9, Riga, 1010 Latvia; 7 Department of Economics, University of Potsdam, August-Bebel-Straße 89, 14482 Potsdam, Germany; 8 Department for Epidemiology and Biostatistics, National Institute for Health Development, Hiiu 42, 11619 Tallinn, Estonia; 9 Institute – European Observatory of Health Inequalities, Calisia University, Nowy Swiat 4, 62-800 Kalisz, Poland; 10 Health Promotion Foundation, Mszczonowska 51, 05-830 Nadarzyn, Poland; 11 Centre for Addiction and Mental Health, Institute for Mental Health Policy Research, 33 Ursula Franklin Street, Toronto, ON M5S 2S1 Canada; 12 Department of Psychiatry, University of Toronto, 250 College Street, 8Th Floor, Toronto, ON M5T 1R8 Canada; 13 Faculty of Medicine, Institute of Medical Science, University of Toronto, Medical Sciences Building, 1 King’s College Circle, Room 2374, Toronto, ON M5S 1A8 Canada; 14 Campbell Family Mental Health Research Institute, Centre for Addiction and Mental Health, 250 College St., Toronto, ON M5T 1R8 Canada; 15 Dalla Lana School of Public Health, University of Toronto, 155 College Street, Toronto, ON M5T 1P8 Canada; 16 Centre for Addiction and Mental Health, World Health Organization / Pan American Health Organization Collaborating Centre, 33 Ursula Franklin Street, Toronto, ON M5S 2S1 Canada; 17 Center for Interdisciplinary Addiction Research (ZIS), Department of Psychiatry and Psychotherapy, University Medical Center Hamburg-Eppendorf (UKE), Martinistraße 52, 20246 Hamburg, Germany; 18 Program On Substance Abuse, Public Health Agency of Catalonia, Program On Substance Abuse & WHO CC, Public Health Agency of Catalonia, 81-95 Roc Boronat St, 08005 Barcelona, Spain

**Keywords:** Alcohol-attributable mortality, Alcohol control policy, Sex, Shift-mean effect, Baltic countries, Poland

## Abstract

**Background:**

The Baltic countries–Lithuania, Latvia and Estonia–are characterized by a high rate of fully alcohol-attributable mortality, compared with Poland. Alcohol control policy measures implemented since 2001 in the Baltic countries included a restriction on availability and an increase in excise taxation, among others. The aim of the current study was to evaluate the relationship between alcohol control policy implementation and alcohol-attributable mortality in the Baltic countries and Poland.

**Methods:**

Alcohol-attributable mortality data for 2001–2020 was defined by codes 100% alcohol-attributable for persons aged 15 years and older in the Baltic countries and Poland. Alcohol control policies implemented between 2001 and 2020 were identified, and their impact on alcohol-attributable mortality was evaluated using an interrupted time-series methodology by employing a generalized additive model.

**Results:**

Alcohol-attributable mortality was significantly higher in the Baltic countries, compared with Poland, for both males and females. In the final reduced model, alcohol control policy significantly reduced male alcohol-attributable mortality by 7.60% in the 12 months post-policy implementation. For females, the alcohol control policy mean-shift effect was higher, resulting in a significant reduction of alcohol-attributable mortality by 10.77% in the 12 months post-policy implementation. The interaction effects of countries and policy tested in the full model were not statistically significant, which indicated that the impact of alcohol control policy on alcohol-attributable mortality did not differ across countries for both males and females.

**Conclusions:**

Based on the findings of the current study, alcohol control policy in the form of reduced availability and increased taxation was associated with a reduction in alcohol-attributable mortality among both males and females.

**Supplementary Information:**

The online version contains supplementary material available at 10.1186/s13011-023-00574-7.

## Introduction

Central and Eastern European countries have the highest alcohol per capita consumption in the European region and, in turn, also have a high alcohol-attributable disease burden [1]. Alcohol consumption has been causally linked to more than 200 diseases, injuries, and other health conditions, including alcoholic liver disease, alcoholic cardiomyopathy, specific cancers, and mental or behavioral disorders [2, 3]. In the case of Estonia, Latvia, and Lithuania–the Baltic countries (BC)–the mortality attributable to alcohol-related chronic liver and cardiovascular diseases (CVD) is particularly high [4–7], which is at least partly explained by specific alcohol consumption patterns (i.e., binge drinking) that characterize these countries, as demonstrated in previous studies [8, 9].

Over the past 30 years, the BC has undergone a societal transformation that has substantially impacted health outcomes and brought significant changes to people’s health behavior. Alcohol consumption is one such example. During the last two decades, alcohol consumption has been similar across the BC, with 10 to 15 L of absolute alcohol being consumed annually per person aged 15 years and older, much higher than the global average [10, 11]. Over the past two decades, some alcohol control policy (ACP) measures have been implemented in the BC [12], which were expected to influence both overall and alcohol-attributable mortality trends in these countries [13]. During those years, significant economic changes took place in the BC, such as the 2008–2009 global financial crisis, which reduced gross domestic product (GDP) and decreased people's ability to purchase certain products, including alcohol. Adopting effective ACP is important to reduce mortality from alcohol-attributable diseases [12]. The most cost-effective policies recommended by the World Health Organization (WHO) (commonly referred to as the “best buys”) include advertising bans, increasing taxes on alcoholic beverages, and reducing the availability of alcohol [14]. Of the three ACP measures, increased taxation and reduced availability are expected to have an immediate impact on alcohol consumption, while advertising bans are expected to have a lagged effect [15, 16].

The implementation of ACP in the BC has been shown to reduce alcohol consumption and change drinking habits [12, 13]. ACP measures, such as a ban on advertising, reduction in the availability of alcohol, and increase in excise taxes on alcohol, implemented in Lithuania have led to significant positive changes in overall mortality and life expectancy [13, 17].

The BC and Poland implemented several ACP measures at various times from 2001 to 2020. In the BC, the number of implemented ACP measures increased around 2008, following the “year of sobriety” in Lithuania and the start of the global economic crisis in all three of the BC, while in Poland, only two ACP enactments were observed [18]. There were also two instances when the alcohol excise tax was reduced (in Poland in 2002 and Estonia in 2019) [16]. In addition to all-cause mortality, which seems to have been the focus of several of the latest studies in Europe [13, 19, 20], alcohol-attributable mortality is undoubtedly the most important criterion for evaluating the impact of ACP measures, as it is necessary for establishing the causal pathway.

Thus, the aim of the current study was to evaluate the relationship between ACP measures (increased taxation and reduced availability) and alcohol-attributable mortality in the BC and Poland.

## Methods

This observational study utilized monthly alcohol-attributable mortality data from 2001 to 2020 (*n* = 240 months) for the BC and from 2001 to 2019 for Poland (*n* = 228 months).

### Alcohol control policy measures

The ACP measure was ascertained via a review of relevant legislation in different countries and following the previous work by Rehm et al. (2022) [16] and Miščikienė et al. (2020) [18]. During the period of investigation, the following 18 ACP implementation time points were evaluated: in Estonia – seven tax increases and one availability reduction, in Latvia – four tax increases and one availability reduction, in Lithuania – two tax increases and two availability reductions, and in Poland – one tax increase. All ACP measures implemented during the analyzed period in the BC and Poland are presented in detail in Table 1. The procedures used to select the ACP measures modeled are described in detail elsewhere [15, 16]. The alcohol excise tax policies were included if the increase resulted in reduced affordability compared to the previous year, and the availability restriction policies were included if they reduced alcohol availability by at least 20%.

Each ACP was assumed to have an immediate impact that lasted 12 months and thus, was assigned a dummy variable, which was set to zero before the implementation of the ACP, then set to one for 12 months after the ACP was implemented, and set back to zero following the 12 months. A combined ACP dummy variable was also considered for each country. It was constructed in the following way: if any of the country's ACPs were active in each month the dummy variable was set to one. Otherwise, this dummy variable was set to zero. This allowed us to collectively assess the impact of all ACPs enacted in all countries simultaneously.

**Table 1:** Policy coding, short explanation, and implementation date for Estonia, Latvia, Lithuania and Poland

Policy number	Short description of alcohol policy	Exact implementation date
**Estonia**		
1. Policy A	Excise tax increases by 10% for all alcoholic beverages	2008–01-01
2. Policy B-1^1^	Excise tax increases by 20% for all alcoholic beverages	2008–07-01
3. Policy B-2^1^	Reduced availability. Off-premise sales nationwide are prohibited between 10 p.m. and 10 a.m	2008–07-14
4. Policy C	Excise tax increases by 10% for all alcoholic beverages	2010–01-01
5. Policy D	Excise tax increases by 15% for all alcoholic beverages	2016–02-01
6. Policy E	Excise tax increases by 10% for all alcoholic beverages	2017–02-01
7. Policy F	Excise tax increases by 45% for wine and by 70% for beer	2017–07-01
8. Policy G	Excise tax increases by 5–20% for all alcoholic beverages	2018–02-01
**Latvia**		
1. Policy A	Retail sales are prohibited from 10 p.m. to 8 a.m	2002–06-14
2. Policy B	Excise tax increases by 11.5 for beer and by 33% for wine	2009–02-01
3. Policy C	Excise tax increases by 7.9% for spirits and by 50.3% for beer	2009–07-01
4. Policy D	Excise tax increases by 7.1–12.5% for wines	2010–02-01
5. Policy E	Excise tax increases by 9–12% for all alcoholic beverages	2019–03-01
**Lithuania**		
1. Policy A	Excise tax increases by 10% for beer and by 20% for other beverages, other implemented policies included banning alcohol advertising on TV and radio during daytime and increased penalties for drunk driving	2008–01-01
2. Policy B	Availability restrictions such as banned off-premise sales from 10 p.m. to 8 a.m	2009–01-01
3. Policy C	Excise tax increases by 23% for ethyl alcohol, by 92–94% for intermediate products, and by 111–112% for wines and beer	2017–03-01
4. Policy D	Reduced availability by increasing the legal minimum age to 20 years (with additional enforcement criteria such as a request for an ID upon purchase if a customer appears to be younger than 25); and reduced off-premise sales hours to 8 pm-10 am Monday-Saturday and on Sundays till 3 p.m.; also, a near full alcohol advertising ban was implemented	2018–01-01
**Poland**		
1. Policy A	Excise tax increases by 9–16% for all alcoholic beverages	2009–03-01

### Mortality data

The sex-specific monthly alcohol-attributable mortality data were obtained by request for Lithuania from Statistics Lithuania and the State Register of Deaths and its Causes at the Institute of Hygiene, for Latvia from the Center for Disease Prevention and Control, for Estonia from the Estonian Cause of Death Registry, and for Poland from the National Statistical Office. The mortality data were obtained for individuals aged 15 years and older.

In total, 27 alcohol-attributable causes of death (five main and 22 additional) based on 10^th^ Revision International Classification of Diseases (ICD-10) codes [21] were selected (Table 2). The specified causes of death are those that are fully attributable to alcohol with an alcohol-attributable fraction (AAF) of 100%, where AAF denotes the proportion of a certain disease category that would not have occurred had there been no alcohol consumption [22].

**Table 2:** The fully alcohol-attributable causes deaths according to the 10^th^ Revision International Classification of Diseases

Main 5 deaths categories	Additional 22 deaths categories
1. mental and behavioral disorders due to the use of alcohol (F10),	1. chronic hepatitis (K73),
	2. alcohol-induced pseudo-Cushing’s syndrome (E24.4),
	3. degeneration of the nervous system due to alcohol (G31.2),
2. accidental poisoning by and exposure to alcohol (X45),	
	4. alcoholic polyneuropathy (G62.1),
	5. alcoholic myopathy (G72.1),
3. alcoholic liver disease (K70),	6. alcoholic gastritis (K29.2),
	7. alcohol-induced acute pancreatitis (K85.2),
4. liver cirrhosis (K74),	8. alcohol-induced chronic pancreatitis (K86.0),
	9. maternal care for suspected damage to the fetus from alcohol (O35.4),
5. alcoholic cardiomyopathy (I42.6),	
	10. fetus and newborn affected by maternal use of alcohol (P04.3),
	11. fetal alcohol syndrome (dysmorphic) (Q86.0),
	12. the finding of alcohol in the blood (R78.0),
	13. toxic effect of alcohol (T51),
	14. intentional self-poisoning by and exposure to alcohol (X65),
	15. poisoning by and exposure to alcohol, undetermined intent (Y15),
	16. evidence of alcohol involvement determined by blood alcohol level (Y90),
	17. evidence of alcohol involvement determined by the level of intoxication (Y91),
	18. blood-alcohol and blood-drug test (Z04.0),
	19. alcohol rehabilitation (Z50.2),
	20. alcohol abuse counseling and surveillance for alcohol use disorder (Z71.4),
	21. alcohol use (Z72.1),
	22. family history of alcohol abuse (Z81.1)

The BC and Poland used ICD-10 codes and the same death coding principles, however, the procedures have slightly changed over the study period and varied by country. A death certificate was issued by physicians in each of the study countries both in outpatient and inpatient health institutions. When recording the cause of death of a deceased person on the death certificate, the physicians relied on the information contained in the outpatient and inpatient medical records. Only those codes on the death certificates that were indicated as the main cause of death were included in the current analysis.

### Control variables

The economic recession was controlled for in each model and was defined by a decrease in GDP based on purchasing power parities (GDP-PPP) using data from OECD [10]. The respective variable was country-specific, as the economic recession affected each of the countries at slightly different time points (see Supplementary material, Annex 1, Table 1S).

### Statistical analyses

For all four countries, monthly time series consisting of age-standardized alcohol-attributable mortality rates per 100,000 population were analyzed. The proportion of alcohol-attributable deaths among total deaths was estimated as a percentage. Age-standardization was done using the European standard population in 2013 for those 15 years and older [23]. Statistical significance was set at *p* < 0.05. All statistical analyses were performed using R version 3.6.3 [24].

To test our hypothesis that ACP was associated with a reduction in alcohol-attributable mortality in BC and Poland, we performed interrupted time-series analyses by employing a generalized additive model (GAM) for both males and females [25]. All GAM models controlled for the economic recession using a dummy coded variable, coded as 1 during the months affected by the recession, and 0 for all other months. Countries were included and represented by a categorical variable, with Poland as the reference category. That is, the country of Poland was assigned a value of 0, and Estonia, Latvia and Lithuania were assigned values of 1, 2, and 3, respectively. Therefore, the coefficients of country effects are interpreted with respect to Poland. The log-transformed age-standardized alcohol-attributable mortality rates were approximately normally distributed, allowing for the use of linear models, and easily transformed into percentage change by exponentiating the coefficients, subtracting one from this number, and multiplying by 100 [26]. Seasonality was adjusted by adding smoothing splines representing monthly and yearly patterns. Residuals were examined with plots of the autocorrelation function and partial autocorrelation function to determine the autoregressive and moving average series orders (see Supplementary material, Annex 2).

For each sex, we presented a full model and a reduced model. Included in the full model were, in addition to the outcome, the linear time trend, ACP, economic recession, countries and the interactions between ACP and each of the countries, as well as the smooth terms. The outcome, alcohol-attributable mortality, was log-transformed to stabilize variance over time. Akaike Information Criterion (AIC) and R-squared were used to assist with selecting the most appropriate model [27]. A lower AIC value indicates a better fit; as such, the model with the lowest AIC was selected. The full model was further optimized to a reduced model by removing non-significant covariates if their inclusion did not improve model fit (as per the AIC or R-squared). Lastly, Chi-square difference tests were used to evaluate whether the full model fit significantly better than the reduced model [28]. All GAM models were conducted using the “mgcv” package in R [29].

To further investigate which ACPs were most effective, interventional Autoregressive Integrated Moving Average (ARIMA) models were developed to estimate the effect of individual ACP intervention while controlling for autocorrelation. ARIMA models were fitted using standard techniques, with AIC as an indicator of model fit. The seasonality was set to be 12 months due to the monthly data.

For each country and policy pair, a seasonal ARIMA model with exogenous variables was estimated (this is denoted as a SARIMAX(p,d,q)(P,D,Q)_12_ model, with specific values of (p,d,q) and (P,D,Q) selected to obtain the best fit to the data). Corrected Akaike’s Information Criterion (AICc) was used to evaluate the quality of the models, with lower values of AICc indicating a better fit to the data. The main parameter related to the impact of a given policy is the coefficient of the exogenous dummy variable corresponding to that specific policy (reported as the Policy effect coefficient). Assuming the effect is significant, a positive policy effect coefficient indicates an increase in mortality in the months following the policy’s implementation, while a negative value implies a congruent decrease in mortality [30]. All ARIMA models were done using the “forecast” package in R [31].

## Results

**Fig. 1:**
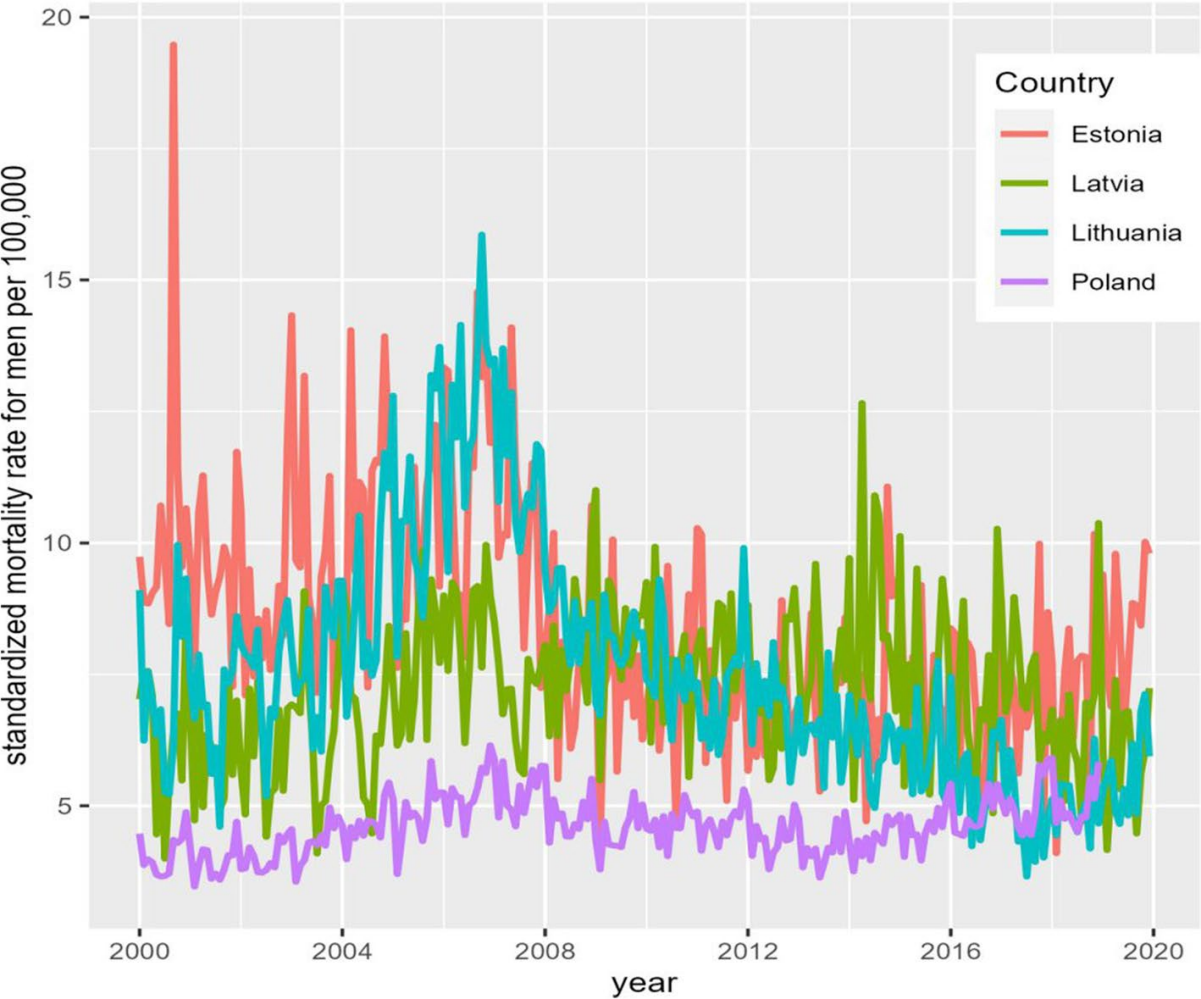
Age-standardized alcohol-attributable mortality in the Baltic countries and Poland among males from 2001 to 2020 (monthly data)

**Fig. 2:**
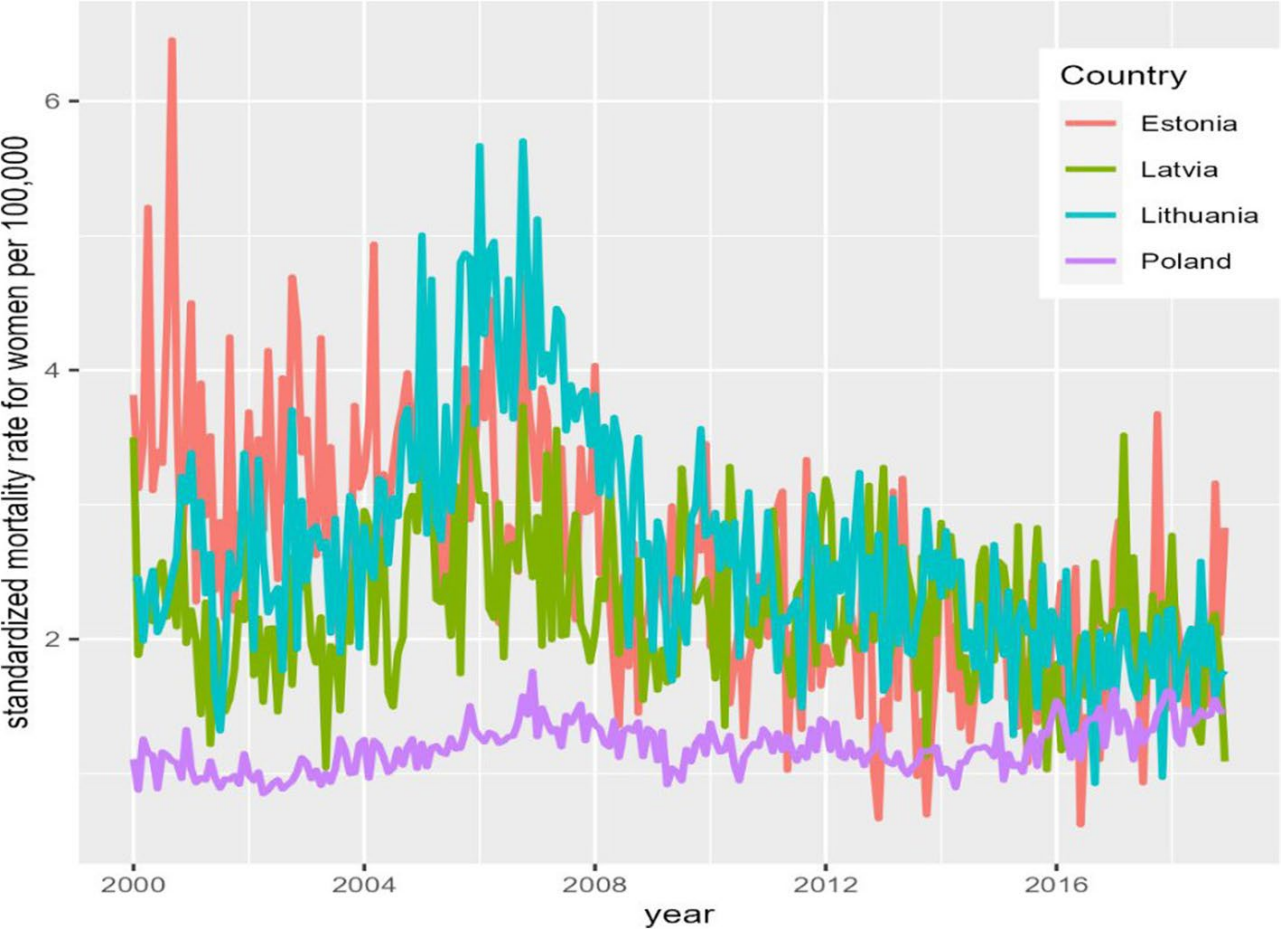
Age-standardized alcohol-attributable mortality in the Baltic countries and Poland among females from 2001 to 2020 (monthly data)

Figures 1 and 2 show the age-standardized alcohol-attributable mortality rates for males and females, respectively, over time. Both time series had some evidence of seasonal variation and non-linear trends over time.

During the study period, the average age-standardized alcohol-attributable mortality rate for males was 8.48 per 100,000 population in Estonia, 7.20 per 100,000 population in Latvia, 7.57 per 100,000 population in Lithuania, and 4.61 per 100,000 population in Poland. The age-standardized alcohol-attributable mortality rate for females was substantially lower compared to males in all four countries: 2.55, 2.21, 2.63, and 1.20 per 100,000 population in Estonia, Latvia, Lithuania, and Poland, respectively.

From 2001 to 2020, the average proportion of alcohol-attributable deaths of total deaths among males was 4.1% in Estonia, 3.1% in Latvia, 3.3% in Lithuania, and 2.5% in Poland. The proportion of alcohol-attributable deaths of total deaths among females was lower and accounted for 2.3%, 1.7%, 2.1%, and 1.1% in Estonia, Latvia, Lithuania, and Poland, respectively.

### Effects of countries

For males, both the “full” model and “reduced” model showed that, on average, Estonia, Latvia, and Lithuania all had higher alcohol-attributable mortality rates than Poland. The reduced model showed that Estonia had an 83.68% (exp(0.616)-1) higher alcohol-attributable mortality rate than Poland, Latvia had a 58.10% higher rate, and Lithuania had a 59.35% higher rate. The impact of ACP did not differ by country, as the interaction effects of countries with ACP in the full model were not statistically significant (Table 3).

**Table 3:** Effect of combined alcohol control policies on alcohol-attributable mortality rates among males in the Baltic countries and Poland

	**Full model**				**Reduced model**			
**Row name**	**Estimate**	**Std. Error**	**95% CI**	***p* value**	**Estimate**	**Std. Error**	**95% CI**	***p* value**
(Intercept)	1.460	0.013	(1.435,1.485)	< 0.001	1.46	0.013	(1.435,1.485)	< 0.001
Estonia	0.624	0.02	(0.585,0.663)	< 0.001	0.608	0.019	(0.571,0.645)	< 0.001
Latvia	0.443	0.019	(0.406,0.48)	< 0.001	0.458	0.018	(0.423,0.493)	< 0.001
Lithuania	0.474	0.02	(0.435,0.513)	< 0.001	0.466	0.018	(0.431,0.501)	< 0.001
Policy	-0.085	0.061	(-0.205,0.035)	0.164	-0.079	0.021	(-0.12,-0.038)	< 0.001
Recession	-0.021	0.032	(-0.084,0.042)	0.513				
Estonia: Policy	-0.046	0.066	(-0.175,0.083)	0.487				
Latvia: Policy	0.088	0.067	(-0.043,0.219)	0.191				
Lithuania: Policy	-0.024	0.067	(-0.155,0.107)	0.716				

R-squared 0.645 for the full model and 0.642 for the reduced model; there are no statistically significant differences between the two models (*P* = 0.05)

For females, similar effects were observed. All three of the BC had higher age-standardized alcohol-attributable mortality rates than Poland: Estonia had a rate that was 104.01% higher, Latvia had a rate that was 84.04% higher, and Lithuania had a rate that was 105.85% higher (Table 4). The interaction effects between countries and ACP in the full model were not statistically significant in the model for females, indicating that the association between ACP and alcohol-attributable mortality rates was not significantly different across countries.

**Table 4:** Effect of combined alcohol control policies on alcohol-attributable mortality rates among females in the Baltic Countries and Poland

	**Full model**				**Reduced model**			
**Row name**	**Estimate**	**Std. Error**	**95% CI**	**p value**	**Estimate**	**Std. Error**	**95% CI**	**p value**
(Intercept)	0.138	0.018	(0.103,0.173)	< 0.001	0.141	0.017	(0.108,0.174)	< 0.001
Estonia	0.728	0.027	(0.675,0.781)	< 0.001	0.713	0.025	(0.664,0.762)	< 0.001
Latvia	0.61	0.026	(0.559,0.661)	< 0.001	0.61	0.025	(0.561,0.659)	< 0.001
Lithuania	0.716	0.026	(0.665,0.767)	< 0.001	0.722	0.025	(0.673,0.771)	< 0.001
Policy	-0.054	0.082	(-0.215,0.107)	0.511	-0.114	0.028	(-0.169,-0.059)	< 0.001
Recession	0.026	0.043	(-0.058,0.11)	0.55				
Estonia: Policy	-0.107	0.088	(-0.279,0.065)	0.227				
Latvia: Policy	-0.054	0.09	(-0.23,0.122)	0.553				
Lithuania: Policy	-0.023	0.09	(-0.199,0.153)	0.802				

R-squared 0.608 for the full model and 0.609 for the reduced model; there are no statistically significant differences between the two models (*P* = 0.48)

### Effects of policy

In the final reduced model for males, ACP had a significant effect on the alcohol-attributable mortality rate (-0.079 (95% CI -0.120, -0.038; *p* < 0.001)), which translates to a reduction of 7.60%. Given the different populations in the countries, this corresponds to an average effect of about 29, 39, 41, and 650 alcohol-attributable deaths avoided for Estonia, Latvia, Lithuania, and Poland, respectively, within one year after introducing a policy. As per the non-significant interaction term, the ACP had a similar impact in all four countries (Table 3).

For the final reduced model for females, the ACP mean-shift effect was notably higher -0.114 (95% CI -0.169, -0.059; *p* < 0.001)), which translates to a 10.77% reduction in alcohol-attributable mortality rates. This corresponds to average effects of around 14, 19, 28, and 291 deaths avoided within one year of policy implementation for Estonia, Latvia, Lithuania, and Poland, respectively (Table 4). Similar to males, the impact of APC and the alcohol-attributable mortality rate among females did not differ by country.

The ARIMA models for individual policies (see Table 1S in Annex 1) indicated that most policies were associated with a decrease in alcohol-attributable mortality rates. The 2009 and 2019 tax increases were significantly associated with a reduction in the alcohol-attributable mortality rate among females in Latvia.

## Discussion

Over the past two decades, age-standardized alcohol-attributable mortality rates for both males and females have decreased significantly in most countries of the WHO European Region, but there are still clear differences between individual European regions, with Eastern Europe and the BC standing out among them [32]. During 2001–2020, alcohol-attributable deaths in the BC and Poland varied between 2.5% and 4.1% of all causes of death for males and between 1.1% and 2.3% for females. This difference between males and females is likely dependent on the fact that the number of males who abuse alcohol in the countries under investigation is much higher than females, that males consume alcoholic beverages more often than females, and the amount of alcohol they drink is higher, which causes significantly greater harm of alcohol consumption [33, 34].

During 2001–2020, the BC had a higher level of alcohol consumption and alcohol-attributable mortality than Poland. In recent years the gap in alcohol consumption between the BC and Poland has declined [35]. However, compared to Poland, the alcohol-attributable mortality rate and proportions of alcohol-attributable deaths among all deaths in the BC remained high.

The different alcohol-attributable mortality rates in the BC and Poland could be partially related to differences in the coding of causes of death. A truly refined coding of the cause of death depends on anamnestic data, lifestyle, the clinical manifestation of existing diseases or pathological conditions, refined clinical diagnosis through various laboratory or instrumental tests, and after death through a pathoanatomical examination. Over the past two decades, the frequency of post-mortem examinations has decreased significantly, reaching only 10% in Lithuania in 2020, making it difficult to determine the exact cause of death [36]. Most physicians judge the final cause of death based on clinical findings or pre-existing conditions that were recently diagnosed. This may introduce some uncertainty into the assessment of the actual cause of death, especially when explaining rarer clinical conditions or causes of death. It should be noted that in our study we examined 27 fully alcohol-attributable causes of death, out of which five main causes accounted for more than 90% of all fully alcohol-attributable deaths in the BC and Poland. According to Rehm and colleagues, many more cause-of-death codes could be attributed to alcohol [22], such as partially alcohol-attributable or injuries [22, 37]. A previous study found that there has been a decline in Lithuanian male and female mortality from cardiovascular diseases over the past two decades, especially since 2008–2009 when ACPs were implemented [6].

Our study indicated that ACP was associated with a reduction in alcohol-attributable mortality by 7.60% on average among males within 12 months after the implementation of the ACP. The impact of ACP measures on alcohol-attributable mortality among females amounted to an average of 10.77% in the 12 months post-implementation. There are several explanations for why ACP had a greater impact on alcohol-attributable mortality rates among females in the BC compared to their male counterparts. For instance, in 2020, more than 75% of the population consuming harmful amounts of alcohol were males [33]. Males are diagnosed with alcohol use disorders four to five times more often than females, males consume more alcohol per drinking episode on average and are more prone to acute behavioral problems related to alcohol use compared to females [38]. However, in females, problematic alcohol consumption develops faster due to bio-physiological processes [39].

Given the ecological study design, it is important to note that causality cannot be established for the associations found in the current study. However, given the different time points of ACP implementation, the multiple control via other countries, and the similarity of effect sizes irrespective of time and countries of implementation make alternative explanations relatively implausible [40].

The interaction effects of countries with ACP were not statistically significant in the full model, indicating that the ACP impacts did not differ by country for both males and females. Similar findings were found when the association between ACP and all-cause mortality was evaluated in the BC and Poland [20]. Taxation increases and availability restrictions had an effect in all countries, on average significantly reducing the age-standardized all-cause mortality rate among males [20]. Other studies have also shown that a significant increase in alcohol excise duty is necessary to achieve rapid results in reducing alcohol-attributable mortality [41–43]. The positive changes in life expectancy over the past fifteen years were caused by a decrease in mortality from external causes of death, cardiovascular diseases, and alcohol-induced disorders [44, 45]. Changes in mortality from external causes of death among females had a lower association with alcohol use and therefore were less influenced by ACP [44].

The WHO recommends raising alcohol taxes to an appropriate level as one of the most effective ways to reduce alcohol-attributable mortality [46]. Thus, the most effective ACP interventions, which led to an immediate change in both overall mortality and alcohol-attributable mortality, were the WHO's so-called "best buys", and were mainly driven by significant increases in alcohol excise taxes [47, 48].

### Limitations

There are a few limitations of the current study that must be acknowledged. First, as mentioned above, the assessment of causality is limited due to the ecological nature of the study. Second, the alcohol-attributable mortality rate may have been reduced in individual countries because of possible ethical principles in capturing other death codes not attributable to alcohol. Third, in some of the countries assessed, the low proportion of autopsies may have caused problems in verifying the diagnosis of death and the possible causes of death associated with alcohol exposure (the percentage of autopsies in the BC decreased 2–3 times over the last two decades). Fourth, over the last two decades, it has not been possible in the countries under investigation to accurately assess compliance with ACP. Fifth, other smaller groups of alcohol-attributable deaths may have brought some changes in the assessment of alcohol-attributable mortality and coding inaccuracies (in the Polish alcohol-related mortality database, among the 22 additional alcohol-related causes of death, only ten alcohol-related cause-of-death codes were completed).

## Conclusions

Alcohol-attributable mortality was significantly higher in Estonia, Latvia and Lithuania, compared with Poland. Based on the findings of the current study, alcohol control policy in the form of reduced availability and increased taxation was associated with reduced alcohol-attributable mortality among both males and females. Further studies analyzing the effect of alcohol control policies on alcohol-attributable mortality in multiple countries are needed.

### Supplementary Information

**Additional file 1: Annex 1.** **Table 1S.** Single-policy models for Baltic countries and Poland by sex and alcohol control policies.

**Additional file 2: Annex 2. **ACF and PACF curves.

## Data Availability

The data that support the findings of this study are not openly available due to reasons of sensitivity and are available from the corresponding author upon reasonable request. Data are located in controlled access data storage at the Health Research Institute of the Lithuanian University of Health Sciences.

## Abbreviations

AAM: Alcohol-attributable mortality
ACP: Alcohol control policy
BC: Baltic countries
