# Supplementary material for: Alcohol-attributable mortality and alcohol control policy in the Baltic Countries and Poland in 2001–2020: an interrupted time-series analysis
Source: Subst Abuse Treat Prev Policy. 2023 Nov 9;18:65. doi: 10.1186/s13011-023-00574-7 (PMC10636906; doi:10.1186/s13011-023-00574-7)
Supplement: Supplementary file 1 — Additional file 1: Annex 1. Table 1S. Single-policy models for Baltic countries and Poland by sex and alcohol control policies. [file 13011_2023_574_MOESM1_ESM.docx]

**Annex 1**

**Table 1S**: Single-policy models for Baltic countries and Poland by sex and alcohol control policies.

| **Sex** | **Policy code** | **SARIMA model order (AICc)** | **Policy effect coefficient (95% CI)** |
| --- | --- | --- | --- |
| **Estonia** |  |  |  |
| Male | Policy A | (0,1,2)(1,0,0)12 (953.899) | 0.7222 (-0.7105, 2.155) |
|  | Policy B | (0,1,2)(1,0,0)12 (953.91) | -0.7014 (-2.108, 0.7053) |
|  | Policy C | (0,1,2)(1,0,0)12 (953.777) | -0.7541 (-2.174, 0.666) |
|  | Policy D | (0,1,2)(1,0,0)12 (954.502) | -0.4309 (-1.863, 1.001) |
|  | Policy E | (0,1,2)(1,0,0)12 (954.825) | -0.1214 (-1.539, 1.296) |
|  | Policy F | (0,1,2)(1,0,0)12 (953.934) | -0.6913 (-2.1, 0.7179) |
|  | Policy G | (0,1,2)(1,0,0)12 (954.814) | 0.1456 (-1.294, 1.585) |
|  |  |  |  |
| Female | Policy A | (1,1,3)(1,0,0)12 (532.073) | 0.1269 (-0.4028, 0.6566) |
|  | Policy B | (1,1,3)(1,0,0)12 (531.531) | -0.2365 (-0.7686, 0.2955) |
|  | Policy C | (1,1,3)(1,0,0)12 (530.899) | 0.3232 (-0.2142, 0.8605) |
|  | Policy D | (1,1,3)(1,0,0)12 (532.118) | -0.1129 (-0.6426, 0.4168) |
|  | Policy E | (1,1,3)(1,0,0)12 (531.656) | -0.2156 (-0.7439, 0.3128) |
|  | Policy F | (1,1,3)(1,0,0)12 (532.291) | -0.00415 (-0.5372, 0.5289) |
|  | Policy G | (1,1,3)(1,0,0)12 (531.919) | 0.166 (-0.3667, 0.6986) |
|  |  |  |  |
| **Latvia** |  |  |  |
| Male | Policy A | (0,1,2) (824.794) | -0.1672 (-1.226, 0.8912) |
|  | Policy B | (0,1,2) (823.812) | 0.5453 (-0.4844, 1.575) |
|  | Policy C | (0,1,2) (822.903) | 0.7382 (-0.2802, 1.757) |
|  | Policy D | (0,1,2)(2,0,0)12 (824.673) | 0.2766 (-0.7111, 1.264) |
|  | Policy E | (0,1,2) (824.763) | -0.196 (-1.274, 0.8822) |
|  |  |  |  |
| Female | Policy A | (2,1,1)(0,0,1)12 (368.119) | -0.1479 (-0.5506, 0.2549) |
|  | Policy B | (2,1,1)(0,0,1)12 (368.615) | 0.02536 (-0.3663, 0.417) |
|  | Policy C | (2,1,1)(0,0,1)12 (363.986) | -0.4172 (-0.7907, -0.04373)* |
|  | Policy D | (2,1,1)(0,0,1)12 (368.467) | -0.08034 (-0.4687, 0.308) |
|  | Policy E | (2,1,1)(0,0,1)12 (363.718) | -0.4424 (-0.8331, -0.05174)* |
|  |  |  |  |
| **Lithuania** |  |  |  |
| Male | Policy A | (0,1,1)(2,0,0)12 (685.155) | 0.4117 (-0.4509, 1.274) |
|  | Policy B | (0,1,1)(2,0,0)12 (683.741) | -0.6768 (-1.535, 0.1819) |
|  | Policy C | (0,1,1)(2,0,0)12 (685.969) | -0.1067 (-0.9792, 0.7658) |
|  | Policy D | (0,1,1)(2,0,0)12 (685.582) | -0.2986 (-1.174, 0.577) |
|  |  |  |  |
| Female | Policy A | (2,1,1)(2,0,0)12 (372.718) | -0.05174 (-0.4752, 0.3718) |
|  | Policy B | (2,1,1)(2,0,0)12 (372.518) | 0.1071 (-0.3068, 0.5211) |
|  | Policy C | (2,1,1)(2,0,0)12 (371.879) | -0.1982 (-0.6063, 0.2099) |
|  | Policy D | (2,1,1)(2,0,0)12 (372.766) | 0.01977 (-0.3942, 0.4338) |
|  |  |  |  |
| **Poland** |  |  |  |
| Male |  |  |  |
|  | Policy A | (1,0,1)(2,1,0)12 (107.048) | -0.2067 (-0.4463, 0.03298) |
|  |  |  |  |
| Female |  |  |  |
|  | Policy A | (0,1,4)(1,0,0)12 (-326.099) | 0.003929 (-0.08929, 0.09714) |
|  |  |  |  |

^2^ – Poland data is available only for the years 2001-2019.

* -- significant at $\alpha=0.05$
