# Supplementary figures and images for: Alcohol-attributable mortality and alcohol control policy in the Baltic Countries and Poland in 2001–2020: an interrupted time-series analysis
Source: Subst Abuse Treat Prev Policy. 2023 Nov 9;18:65. doi: 10.1186/s13011-023-00574-7 (PMC10636906; doi:10.1186/s13011-023-00574-7)

## **Annex 2**

## ACF and PACF curves

It is the graphs of autocorrelations:
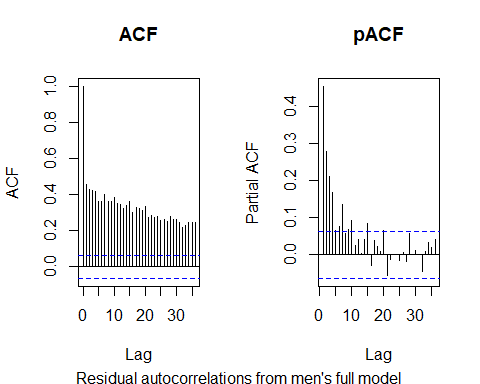

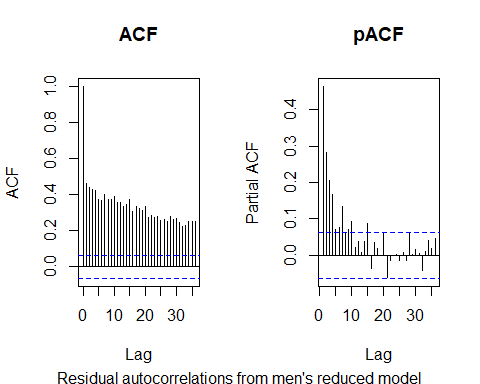

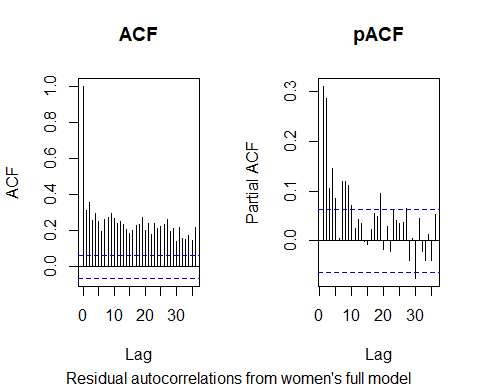

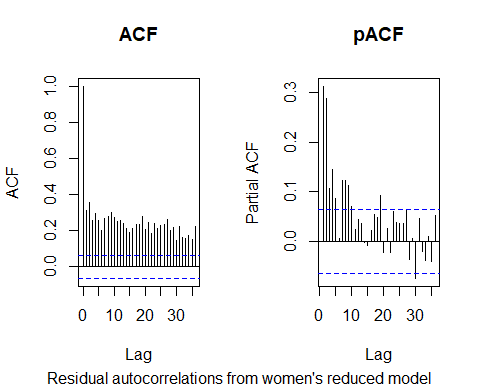

Supplement: Supplementary file 2 — Additional file 2: Annex 2. ACF and PACF curves. [file 13011_2023_574_MOESM2_ESM.docx]
